# Effectiveness and Safety of IPN-20-SENSE LIDOCAINE for Lip Volume Augmentation and/or Redefinition (SMILE Study): A Non-inferiority Randomized Double-Blinded Controlled Study

**DOI:** 10.1007/s00266-024-04398-z

**Published:** 2024-10-14

**Authors:** Nicolas Froget, Izabela Kobylińska, Olga Warszawik-Hendzel, Thomas Guidicelli, Julia Gotlib

**Affiliations:** 1 Clinique Charcot, 51-53 rue Commandant Charcot, 69110 Sainte-Foy-lès-Lyon, France; 2 Medspa, Czarny Dwor 14/7, 80-365 Gdansk, Poland; 3 Department of Dermatology, The Infant Jesus Teaching Hospital, Warsaw Medical University, Koszykowa Street, 82A, 02-008 Warsaw, Poland; 4 Hôpital privé La Casamance, 33 boulevard des Farigoules, 13400 Aubagne, France; 5 15 rue Masséna, 06000 Nice, France

**Keywords:** Hyaluronic acid, Cross-linked, Dermal filler, Lip volume, Stylage, Randomized controlled trial (RCT)

## Abstract

**Background:**

Hyaluronic acid (HA) is a widely used dermal filler for lip augmentation. IPN-20-SENSE LIDOCAINE (Laboratoires VIVACY) is a monophasic gel consisting of cross-linked HA and includes lidocaine hydrochloride for the reduction of injection-associated pain.

**Aims:**

The SMILE study was designed to assess the non-inferiority of IPN-20-SENSE LIDOCAINE compared to HA-RK-Lido in improving aesthetic lip appearance. The secondary objectives were to evaluate the effectiveness and safety of IPN-20-SENSE LIDOCAINE against the chosen similar active control device.

**Patients/Methods:**

This was a prospective, multicenter, double-blinded, randomized active controlled parallel group study undertaken in two investigational sites between May 2021 and July 2022 (14 months). The primary endpoint of this study was the proportion of subjects reporting an improvement on the Global Aesthetic Improvement Scale (GAIS) 3 months after treatment initiation.

**Results:**

Regarding the primary endpoint, the difference between the treatment arms in the proportion of improved subjects was +7.0 [-2.2; 17.7]. The lower limit of this 90% CI, being above the non-inferiority margin of -15 and below zero, demonstrated the non-inferiority of IPN-20-SENSE LIDOCAINE vs. HA-RK-Lido. The secondary outcomes reported by subjects and the blinded live evaluators supported this result. All injection site reactions and device-related adverse events reported in this study were expected, already described and were mostly mild and transient.

**Conclusions:**

The study demonstrated the non-inferiority of IPN-20-SENSE LIDOCAINE to HA-RK-Lido in the aesthetic improvement of the lips. The effect of both treatments decreased over time. Nonetheless, aesthetic improvement was sustained longer in the IPN-20-SENSE LIDOCAINE arm.

**Level of Evidence I:**

This journal requires that authors assign a level of evidence to each article. For a full description of these Evidence-Based Medicine ratings, please refer to the Table of Contents or the online Instructions to Authors www.springer.com/00266.

**Supplementary Information:**

The online version contains supplementary material available at 10.1007/s00266-024-04398-z.

## Introduction

Since the beginning of recorded history, full lips have been associated with youth, beauty, and sensuality in all cultures [1]. Playing a central role in the aesthetic appearance of the lower part of the face, lips are considered as one of the most important parts of facial attractiveness and hence, considered as a focal area of beauty [2, 3]. The upper lip has a significant effect on aesthetic judgement of the face [4]. The relative attractiveness of the lips is determined by their proportions, definition and volume [5].

Non-surgical interventions for lip augmentation are increasing in popularity, and HA is among the most widely used dermal fillers [6]. IPN-20-SENSE LIDOCAINE (Laboratoires VIVACY) is a monophasic gel consisting of cross-linked HA and includes lidocaine hydrochloride for the reduction of injection-associated pain [7]. The product is specifically designed to be injected into and/or around the lips’ mucosa for lip volume augmentation and redefinition.

The SMILE study was a post-market randomized, double-blinded, controlled study designed with the primary objective of assessing the non-inferiority of IPN-20-SENSE LIDOCAINE (intervention) compared to HA-RK-Lido (active control) in improving aesthetic lip appearance as evaluated by the subjects 3 months after treatment initiation. As secondary objectives, several clinical and patient-reported outcomes were collected at 1, 3, 6, 9 and 12 months to evaluate the effectiveness and safety of IPN-20-SENSE LIDOCAINE compared to the chosen active control with regard to subjects and blind live evaluator assessed global aesthetic improvement of the lips, subjects’ satisfaction, pain during injection and any reported adverse events.

## Materials and Methods

The SMILE study was a prospective, multicenter, double-blinded, randomized active controlled parallel group study undertaken in 2 investigational sites specialized in dermatological clinical studies (Contract Research Organization DERMSCAN, in Villeurbanne, France and in Gdansk, Poland) over 14 months, between May 2021 and July 2022.

### Ethics and Regulatory Approvals

This investigation was conducted in compliance with the ethical principles of Good Clinical Practice (GCP), fulfilling the International Organization for Standardization ISO 14155:2020 standard requirements, and in accordance with the Declaration of Helsinki. Informed consent was obtained from each study participant prior to recruitment. The study received favorable ethical approval in each of the participating countries and was registered at ClinicalTrials.gov prior to commencement.

### Population

Subjects’ selection criteria were in line with the instructions for use of both products. Healthy volunteers, of any gender, between the ages of 18 and 65 years, requesting volume augmentation/restoration, and/or outline redefinition of their lips, with attainable expectations and deemed to need it according to the investigators, were considered potentially eligible for inclusion into the study. Participants’ selection criteria were comparable to those used in previous trials that established efficacy of the active control. The full list of inclusion / exclusion criteria is provided in the supplemental material. All participants had to provide a valid written informed consent for participation including agreement to have their facial photographs taken and stored and to follow the study process and follow-up visits in line with the study protocol.

### Intervention and Comparator

The intervention tested in the SMILE study was IPN-20-SENSE LIDOCAINE (manufactured by Laboratoires VIVACY, Archamps, France) while the active comparator was HA-RK-Lido (manufactured by Q-Med AB, Uppsala, Sweden). Both devices are on the market and have European Conformity (CE) marking (since 2009 and 2008, respectively). The comparator device was chosen for its close similarity to the interventional device in terms of clinical, biological and technical characteristics and its proven safety and effectiveness in restoring and augmenting lip volume [8, 9]. IPN-20-SENSE LIDOCAINE is a resorbable, injectable device, consisting of HA gel of non-animal origin, at a concentration of 20 mg/g, cross-linked with BDDE using Inter-Penetrated Networks (IPN^®^)-Like technology. The gel incorporates mannitol as an excipient, together with an anesthetic (lidocaine hydrochloride, 3 mg/g (0.3%) per syringe). HA-RK-Lido is a resorbable, injectable device containing 20 mg/mL cross-linked HA (BDDE) and 3 mg/mL lidocaine hydrochloride. Both products are cross-linked with 1,4-butandioldiglycidyl ether (BDDE), but based on different reticulation techniques, and are monophasic gels with close, but not strictly identical, general rheological properties as assessed in similar conditions of measurements at the frequency of 1 Herz (elastic modulus G’ of 194 Pa versus 174 Pa, viscous modulus of 32 Pa versus 25 Pa, and tangent delta tan *δ* (*G*’’/*G*’) of 0.16 versus 0.14 for IPN-20-SENSE LIDOCAINE and HA-RK-Lido, respectively).

### Study Outcomes

The main research hypothesis for the study was to assess the non-inferiority of IPN-20-SENSE LIDOCAINE compared to HA-RK-Lido with regard to the primary endpoint. The hypothesis for the secondary outcomes was to assess the superiority of the intervention for exploratory purposes. The primary endpoint of this study was the proportion of subjects reporting an improvement on the 5-point Global Aesthetic Improvement Scale (GAIS) (on mirror self-examination), 3 months after treatment initiation. GAIS is a 5-point Likert scale ranging from “Very much improved” to “Worse” (supplemental material) [8, 9]. For the purpose of analysis, any of the first 3 options was considered an indication of improvement.

As secondary endpoints, we assessed the proportion of subjects reporting aesthetic improvement on GAIS following mirror self-examination at 1, 6, 9 and 12 months post-injection, proportion of subjects assessed by the blinded live evaluators to have aesthetic improvement of the lips on GAIS at 1, 3, 6, 9 and 12 months post-injection and FACE-Q™ “Satisfaction with Lips” questionnaire (supplemental material) [10] completed by the subjects before the first injection and at 1, 3, 6, 9 and 12 months post-injection. Prior to commencing the study, all evaluators received training in the use of GAIS and the lips’ surface aspect questionnaires. Participants were also asked to score their pain during injection using an 11-point numeric rating scale (0 to 10) at the first injection and at one month if a touch-up was performed. Additionally, product tolerance was assessed by collection of injection site reactions (ISRs) and adverse events (AEs) throughout the 12-month study follow-up. ISRs were assessed by the blinded live evaluators at each study visit, and by the subjects on daily basis in diaries for 1 month following treatment administration (first and any touch-up injections) using an ISR scoring tool (supplemental material). Subjects were also advised to report any other adverse events occurring at any other timepoint during the study in their diaries.

### Procedures and Follow-Up

Eligible subjects who agreed to participate and provided a valid written informed consent were randomized to either receiving the intervention device (IPN-20-SENSE LIDOCAINE) or the active comparator (HA-RK-Lido). Injection procedures are presented in the supplemental material. Participants were invited back for follow-up appointments at 1, 3, 6, 9 and 12 months post-injection when the primary and secondary outcomes were collected. An optional touch-up was possible at the 1-month follow-up appointment if deemed necessary, according to the subject’s and injector’s opinions. Both the subjects and the live evaluators were blind to the injected device throughout the study. The evaluators used photographs of the subjects taken before the first injection as a reference for comparison and, the same evaluator followed-up the same subjects throughout the study period.

### Randomization and Blinding

Randomization was performed by each investigational site according to randomization lists established by the subcontractor responsible for the statistics before the start of the study with a block size of 8. Subjects were randomized at a 1:1 ratio to receive either the intervention or active control. Randomized subjects were blinded to the administered treatment by using a blindfold during the injection sessions. Live evaluators who saw subjects for follow-up and assessed the aesthetic evolution of their lips using GAIS and the lip surface aspect questionnaire, as well as technicians responsible for capturing photographic images were also blinded to treatment allocation. It was not feasible to blind injectors due to the visual differences in syringes and the injection force required between products, and altering any of the delivery systems would not have been compatible with this phase of study.

### Sample Size

In a previous study assessing the effectiveness of the active control, the estimated lower limit of the two-sided 95% confidence interval (CI) of improvement proportion was 88% [9]. If the intervention was to preserve at least 80% of the comparator’s effect, a non-inferiority margin of 18% would represent 20% (i.e., the intended effect loss) of the lower limit of the two-sided 95% CI of the improvement proportion on GAIS of HA-RK-Lido. A more conservative non-inferiority margin of 15% (rather than 18%) was chosen, which preserves 83% of the effect of HA-RK-Lido. Based on the assumption that the proportion of subjects reporting an improvement following treatment with HA-RK-Lido would be 92%, a non-inferiority margin of -15% and a 1:1 randomization ratio, it was estimated that a minimum of 82 subjects would be required for a 1-sided test at the 5% significance level to detect such margin with 80% power. Assuming a dropout rate of 10% at the primary endpoint (3 months after baseline treatment), it was planned to include a total of 92 subjects in the study.

### Statistical Analysis

Statistical analyses were performed using the SAS^®^ software version 9.4 (SAS Institute, Cary, North Carolina, United States of America). The main non-inferiority comparative analysis of the primary endpoint was performed in the per protocol population, no missing data imputations were necessary for this analysis. Analysis of the main primary effectiveness endpoint was repeated in the full analysis set population according to the treatment group they were randomized into, regardless of receiving the allocated treatment or not.

If non-inferiority was demonstrated, it was planned to analyze the superiority of IPN-20-SENSE LIDOCAINE compared to HA-RK-Lido, defined by the lower limit of the two-sided 90% CI of the difference between the intervention and the comparator in proportions of improved subjects at Month 3 being >0%. The two-sided 90% CI of the between-group difference was obtained using the Newcombe–Wilson method. Proportions of improved subjects were tabulated in each treatment group with two-sided 95% CI and computed using the Clopper–Pearson (exact) method. The overall level of significance for the study was set at 0.05, one-sided for the primary endpoint and two-sided for all other outcomes.

## Results

### Recruitment and Participant Characteristics

**Fig. 1:**
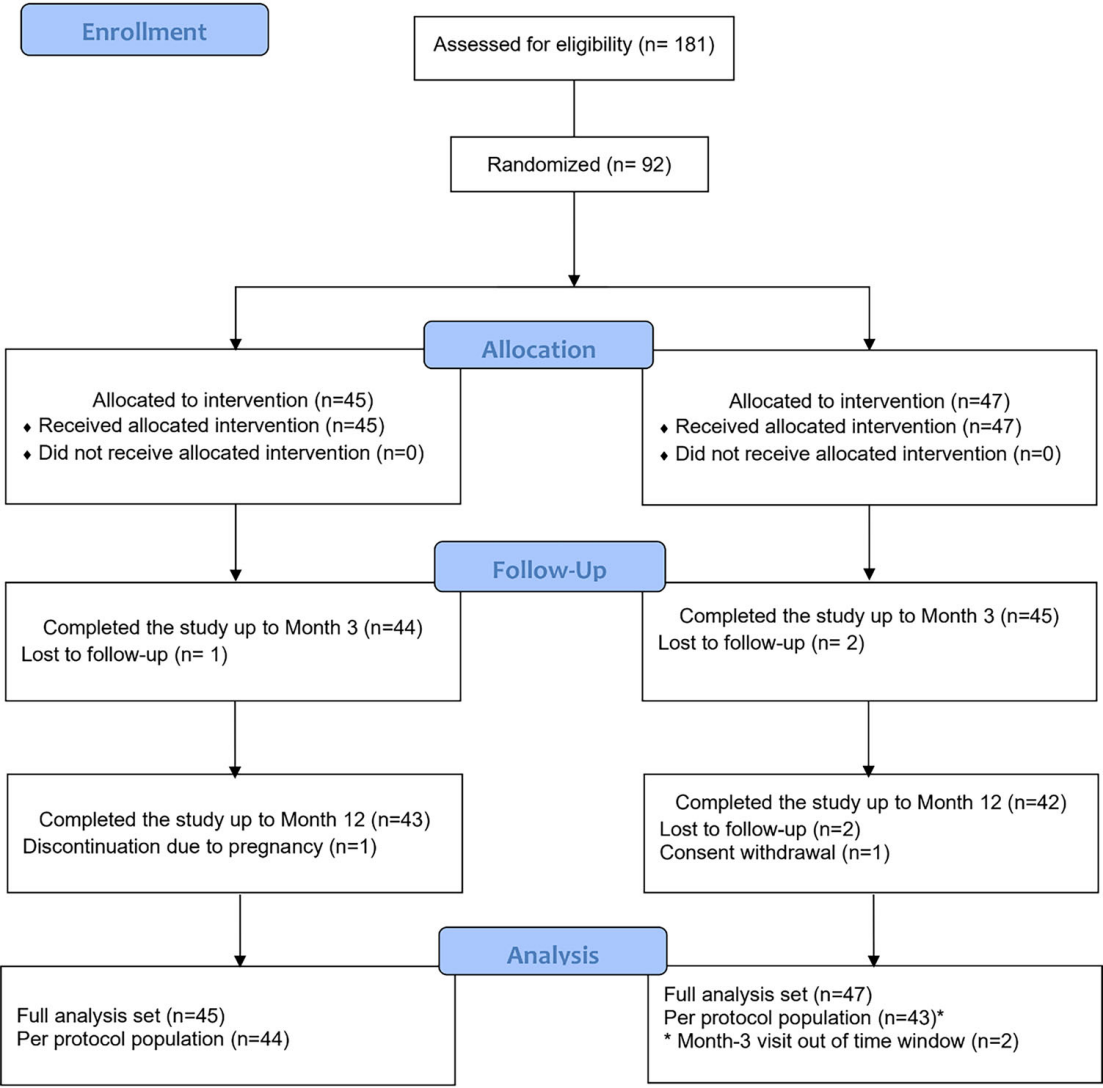
SMILE study CONSORT Flow Diagram

Out of a total of 181 screened subjects, 92 met the inclusion / exclusion criteria and were randomized to either receiving IPN-20-SENSE LIDOCAINE (*n=*45) or HA-RK-Lido (*n=*47). All participants received the treatment they were randomly allocated to. A total of 89 (96.7%) subjects completed the study up-to the 3-month follow-up appointment (44 in the IPN-20-SENSE LIDOCAINE arm and 45 in the HA-RK-Lido arm). The first and last subjects’ 1st visits were in May and July 2021, respectively, and the last subject’s last visit was in July 2022. The flow of participants throughout the study is presented in Fig. 1.

Both study arms were comparable with regard to their demographics and mean FACE-Q™ “Satisfaction with Lips” scores at baseline. Participants’ baseline demographic characteristics and FACE-Q™ “Satisfaction with Lips” are presented in Table 1.

**Table 1:** Participants baseline characteristics—Safety population*

Parameter	IPN-20-SENSE LIDOCAINE (*N=*45)	HA-RK-Lido (*N*=47)
*Sex*		
Male	1 (2.2%)	0 (0.0%)
Female	44 (97.8%)	47 (100.0%)
*Age (years)*		
Mean (SD)	35.2 (12.17)	31.2 (9.34)
Median	32.0	28.0
Q1; Q3	27.0; 43.0	24.0; 36.0
*BMI* (kg/m^2^)		
Mean (SD)	24.94 (4.855)	23.01 (3.887)
*Phototype*		
I	1 (2.2%)	0 (0.0%)
II	8 (17.8%)	12 (25.5%)
III	27 (60.0%)	23 (48.9%)
IV	9 (20.0%)	12 (25.5%)
V	0 (0.0%)	0 (0.0%)
VI	0 (0.0%)	0 (0.0%)
*Smoker*		
Yes	8 (17.8%)	8 (17.0%)
No	37 (82.2%)	39 (83.0%)
*FACE-Q™ “Satisfaction with Lips” score at baseline*		
Mean (SD)	36.2 (12.38)	35.8 (13.27)
Median	37.0	37.0
Q1; Q3	30.0; 42.0	30.0; 47.0

No significant differences in baseline characteristics between groups

*Safety (SAF) population: comprised all the subjects who were included and who received at least one treatment with any of the study devices. Subjects in the SAF population were analyzed according to the received treatment.

*N* Number of subjects with available data; *Q1* First quartile; *Q3* Third quartile; *SD* Standard deviation

### Injection Characteristics

At baseline, all 92 subjects were injected in the upper lip. The lower lip was injected in 86 (93.5%) subjects (43 in each arm), and a total of 48 (53.3%) subjects had a touch-up for either the upper lip, lower lip or both lips at the 1-month follow-up visit. Details of injection characteristics are included in the supplemental material.

#### IPN-20-SENSE LIDOCAINE Effectiveness

##### Primary Outcome

All 44 (100.0%) subjects who had IPN-20-SENSE LIDOCAINE rated the aesthetic appearance of their lips as improved (“Very much improved”, “Much improved” or “Improved”), compared to 40 (93.0%) in the HA-RK-Lido arm (Table 2). The difference and the two-sided 90% confidence interval (CI) of this difference were +7.0 [−2.2; 17.7] with the lower limit of the CI being above the −15% cut-off for demonstrating IPN-20-SENSE LIDOCAINE non-inferiority (Table 2). However, the lower limit of the two-sided 90% CI of this difference was below the 0-cut-off set to demonstrate its superiority. Results from supportive sensitivity analyses in the full analysis set population with and without imputation of missing data were consistent with those obtained in the per protocol population.

**Table 2:** Global aesthetic improvement of the lips assessed by the subjects at 3 months after first injection and in between groups comparative analysis—Primary endpoint—Per-protocol population

Parameter	IPN-20-SENSE LIDOCAINE (*N*=44)	HA-RK-Lido (*N*=43)	Difference IPN-20-SENSE LIDOCAINE minus HA-RK-Lido
*n*	44	43	–
*GAIS score*			
Very much improved	14 (31.8%)	8 (18.6%)	
Much improved	20 (45.5%)	12 (27.9%)	
Improved	10 (22.7%)	20 (46.5%)	
No change	0 (0.0%)	3 (7.0%)	
Worse	0 (0.0%)	0 (0.0%)	
*GAIS improvement*			
Improved subjects^a^	44 (100.0%)	40 (93.0%)	–
95% CI^b^	[92.0; 100.0]	[80.9; 98.5]	–
Difference and 90% CI^c^	–	–	7.0 [−2.2; 17.7]

*CI* Confidence interval; *GAIS* Global aesthetic improvement scale; *N* Number of subjects; *n* Number of subjects with available data

^a^Improvement on the 5-point GAIS corresponds to any of the three following categories “Very much improved”, “*Much improved” and “Improved*”

^b^Clopper–Pearson method

^c^Newcombe–Wilson method with continuity correction

**Fig. 2:**
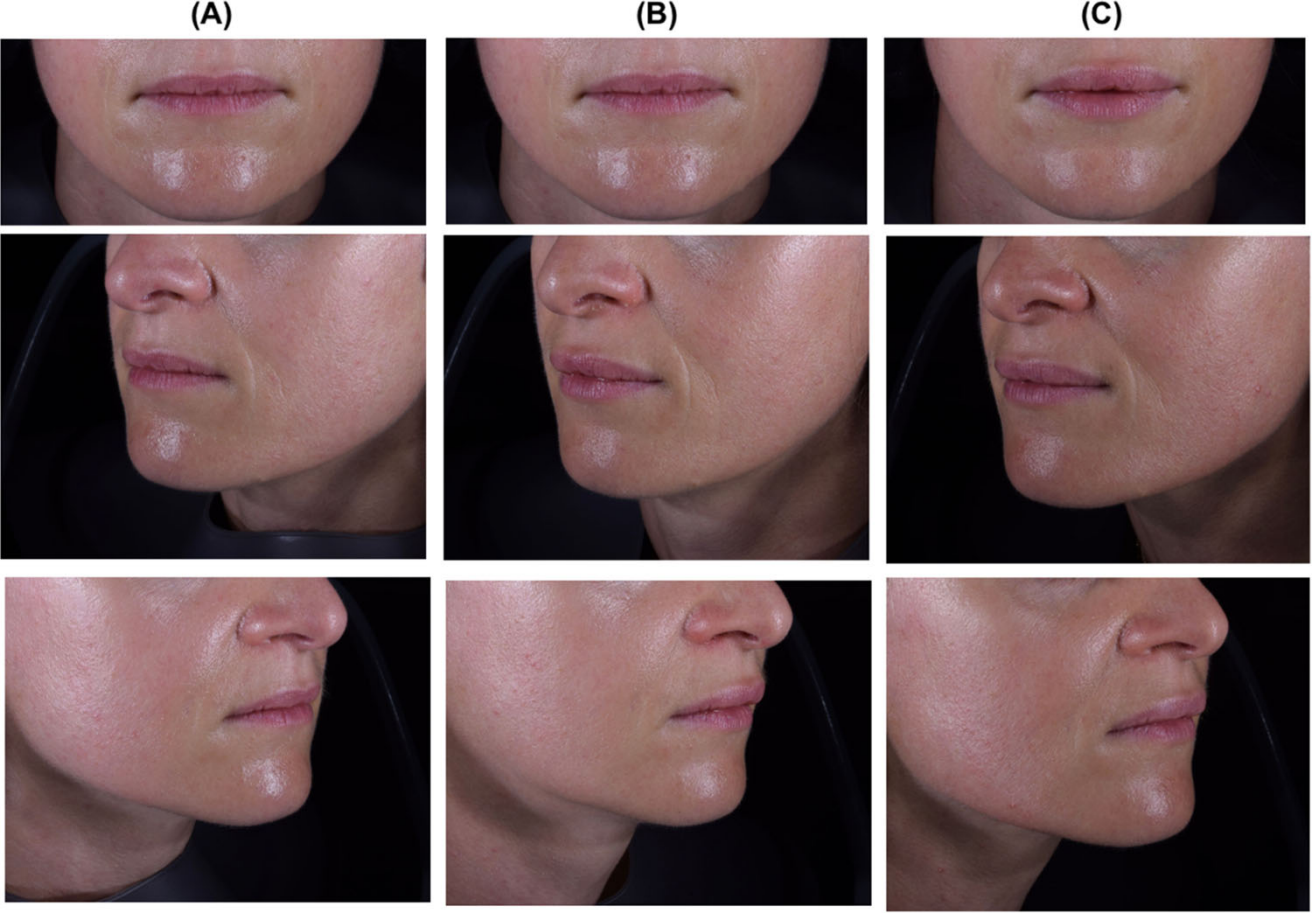
Lip appearance of a 33-year-old woman at (**A**) baseline, and at (**B**) Month 3 and (**C**) Month 12 after injection of IPN-20-SENSE LIDOCAINE. The subject received 0.7 mL in the upper lip and 0.3 mL in the lower lip at first injection and no touch-up injection one month after (not considered necessary by the subject). She rated the overall aesthetic improvement of her lips as “Much improved” at Month 3 and “No change” at Month 12 (and as “Very much improved” and “Much improved” at Months 6 and 9, respectively)

**Fig. 3:**
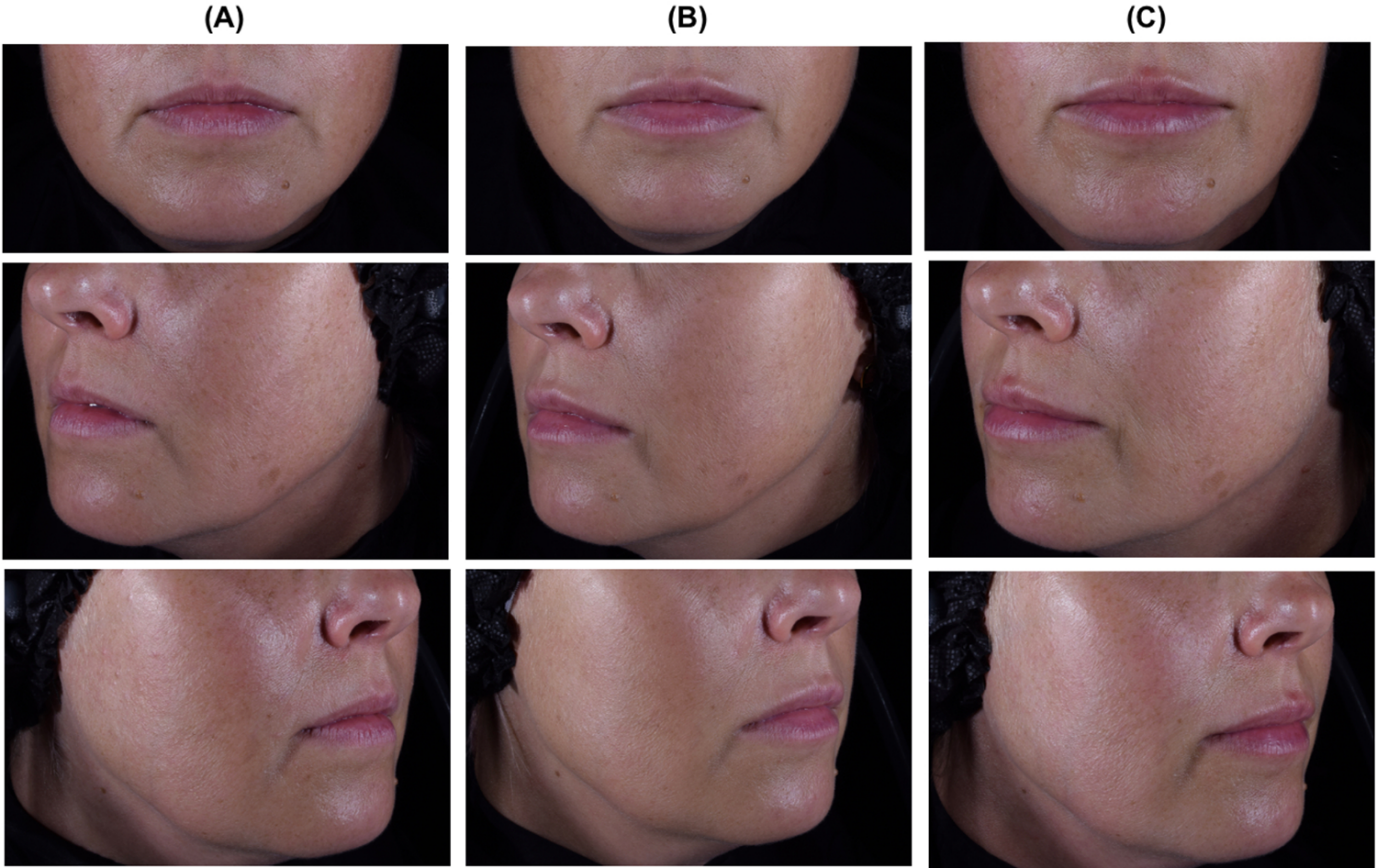
Lip appearance of a 38-year-old woman at (**A**) baseline, and at (**B**) Month 3 and (**C**) Month 12 after injection of IPN-20-SENSE LIDOCAINE. The subject received 0.6 mL in the upper lip and 0.3 mL in the lower lip at first injection and 0.3 mL in the upper lip and 0.1 mL in the lower lip at the touch-up. She rated the overall aesthetic improvement of her lips as “Improved” at Month 3, 6, 9 and 12

Figures 2 and 3 illustrate the aesthetic improvement of the lips in 2 subjects after IPN-20-SENSE LIDOCAINE injection.

##### Secondary Outcomes

**Fig. 4:**
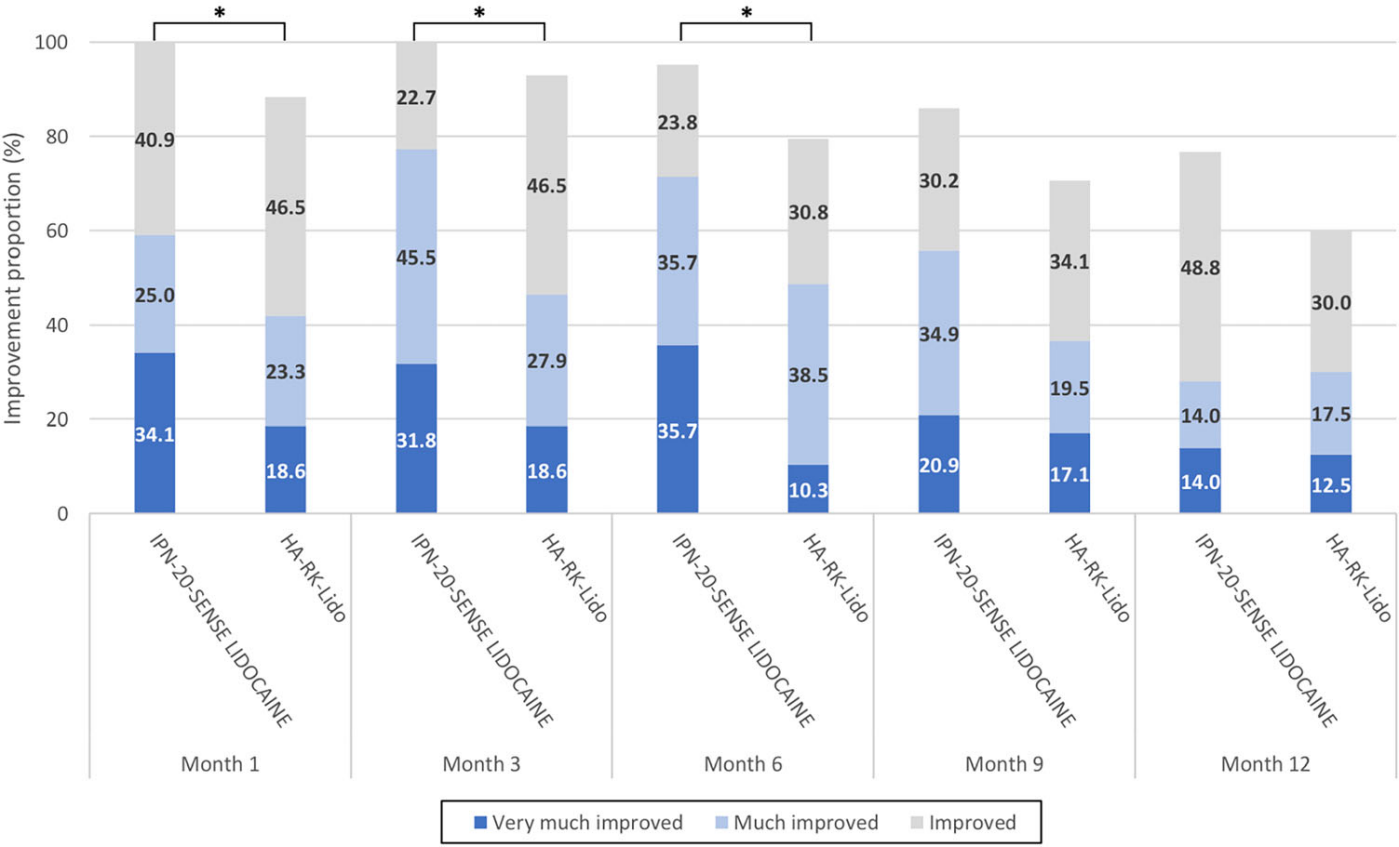
Subjects evaluated Global Aesthetic Improvement Scale at the different follow-up timepoints—Per-protocol population

Subjects’ self-assessment using GAIS at 1 month (before touch-up) and at 6, 9 and 12 months are presented in Table 3 and S1 and Fig. 4. The proportions of improved subjects were higher in the IPN-20-SENSE LIDOCAINE arm at all the assessed timepoints. However, this was only statistically significant at 1 and 6 months (*p*=0.026 and *p*=0.043, respectively).

**Table 3:** Global aesthetic improvement of the lips assessed by the subjects at 1, 6, 9 and 12 months and in between groups comparative analyses—Secondary endpoint—Per-protocol population

Parameter	Month 1^a^		Month 6		Month 9		Month 12	
	IPN-20-SENSE LIDOCAINE (*N*=44)	HA-RK-Lido (*N*=43)	IPN-20-SENSE LIDOCAINE (*N*=44)	HA-RK-Lido (*N*=43)	IPN-20-SENSE LIDOCAINE (*N*=44)	HA-RK-Lido (*N*=43)	IPN-20-SENSE LIDOCAINE (*N*=44)	HA-RK-Lido (*N*=43)
n	44	43	42	39	43	41	43	40
*GAIS improvement*								
Improved subjects^b^	44 (100.0%)	38 (88.4%)	40 (95.2%)	31 (79.5%)	37 (86.0%)	29 (70.7%)	33 (76.7%)	24 (60.0%)
[95% CI]^c^	[92.0; 100.0]	[74.9; 96.1]	[83.8; 99.4]	[63.5; 90.7]	[72.1; 94.7]	[54.5; 83.9]	[61.4; 88.2]	[43.3; 75.1]
Difference in improvement rate^d^	11.6		15.8		15.3		16.7	
[95% CI]^e^	[−0.7; 25.9]		[−0.8; 32.6]		[−4.0; 33.7]		[−4.8; 36.7]	
*p* value^f^	0.026		0.043		0.113		0.155	

Variability was not estimated in the same way for 95% CI of the difference in proportions and statistical test comparing the proportions. This generated intervals that were not consistent with the result of the statistical test, considering that the result was near the significance bound.

*CI* Confidence interval; *GAIS* Global aesthetic improvement scale; *N* Number of subjects; *n* Number of subjects with available data.

^a^Evaluation at Month 1 performed before the touch-up injection (if touch-up performed)

^b^Improvement on the 5-point GAIS corresponds to any of the three following categories “Very much improved”, “Much improved” and “Improved”

^c^Clopper–Pearson method

^d^Difference calculated as [improvement in the IPN-20-SENSE LIDOCAINE arm] minus [improvement in the HA-RK-Lido arm]

^e^Newcombe–Wilson method with continuity correction

^f^Fisher’s exact test

**Fig. 5:**
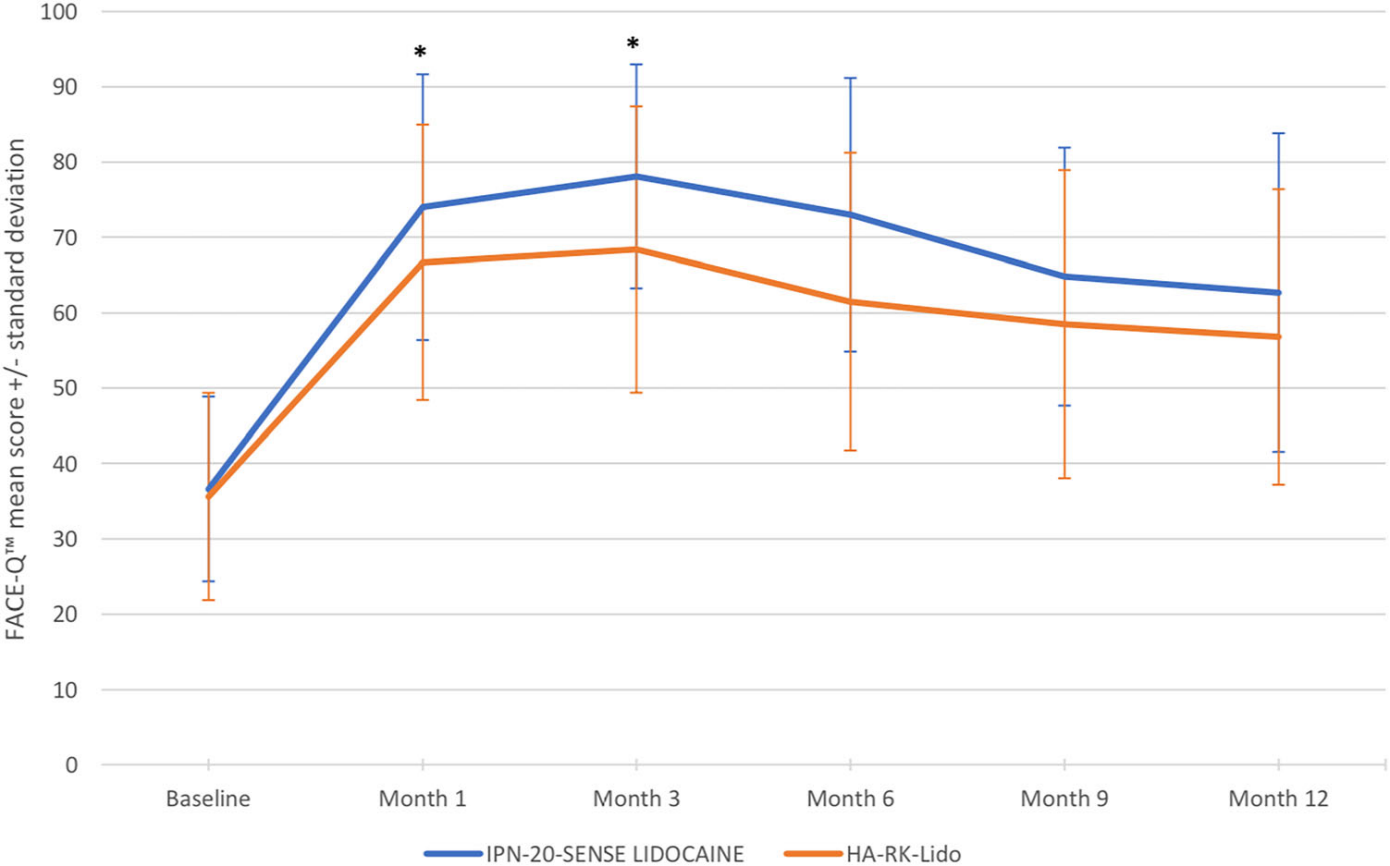
FACE-Q™ score of “Satisfaction with Lips” at the different follow-up timepoints—Per-protocol population

In both arms, the mean FACE-Q™ scores increased rapidly from baseline to Month 3, where it peaked, then decreased slowly from Month 3 to Month 12 but remained higher than baseline. These scores were higher in the IPN-20-SENSE LIDOCAINE group than in the HA-RK-Lido group (Table 4 and Fig. 5).

**Table 4:** FACE-Q™ score of “Satisfaction with Lips” at all timepoints and changes from baseline—Secondary endpoint population

Parameter	Baseline		Month 1^a^		Month 3		Month 6		Month 9		Month 12	
	IPN-20-SENSE LIDOCAINE (*N=*44)	HA-RK-Lido (*N=*43)	IPN-20-SENSE LIDOCAINE (*N=*44)	HA-RK-Lido (*N=*43)	IPN-20-SENSE LIDOCAINE (*N=*44)	HA-RK-Lido (*N=*43)	IPN-20-SENSE LIDOCAINE (*N=*44)	HA-RK-Lido (*N=*43)	IPN-20-SENSE LIDOCAINE (*N=*44)	HA-RK-Lido (*N=*43)	IPN-20-SENSE LIDOCAINE (*N=*44)	HA-RK-Lido (*N=*43)
*n*	44	43	44	43	44	43	42	39	43	41	43	40
*Actual value*												
Mean (SD)	36.6 (12.27)	35.6 (13.76)	74.0 (17.63)	66.7 (18.30)	78.1 (14.85)	68.4 (19.03)	73.0 (18.15)	61.5 (19.76)	64.8 (17.14)	58.5 (20.45)	62.7 (21.15)	56.8 (19.61)
Median	37.0	37.0	76.0	64.0	78.5	64.0	74.5	64.0	64.0	56.0	64.0	57.5
Q1; Q3	30.0; 42.0	28.0; 47.0	61.5; 86.0	53.0; 83.0	64.0; 87.5	56.0; 83.0	59.0; 86.0	45.0; 72.0	56.0; 77.0	47.0; 64.0	47.0; 80.0	45.0; 64.0
*FACE-Q*^TM^* score of “Satisfaction with Lips”—change from baseline*												
Adjusted mean^b^ (SE)			37.8 (2.55)	30.8 (2.58)	41.8 (2.55)	32.5 (2.58)	36.6 (2.83)	26.1 (2.92)	28.2 (2.70)	23.1 (2.77)	26.1 (2.95)	21.2 (3.03)
Adjusted mean of difference^b,c^			7.0		9.3		10.5		5.2		4.9	
[95% CI]^d^			[−0.3;14.2]		[2.1;16.5]		[2.5;18.6]		[−2.5;12.8]		[−3.5;13.3]	
*p* value			0.059		0.012		0.011		0.186		0.250	

*CI* Confidence interval; *MMRM* Mixed model for repeated measurements; *N* Number of subjects; *n* Number of subjects with available data; *Q1* First quartile; *Q3* Third quartile; *SD* Standard deviation; *SE* Standard error.

^a^Evaluation at Month 1 performed before the touch-up injection (if touch-up performed).

^b^Analyzed using a MMRM, including treatment group and timepoint as fixed factors, baseline score as covariate and treatment group by timepoint interaction.

^c^Difference calculated as [change from baseline in the IPN-20-SENSE LIDOCAINE arm] minus [change from baseline in the HA-RK-Lido arm].

^d^Two-sided 95% CI of the between-group difference in mean changes obtained from the contrasts of the treatment factor by time effect.

The mean changes in this score from baseline to 12 months were analyzed and compared using the Mixed Model for Repeated Measurements. The model included treatment group and timepoint as fixed factors, baseline score as covariate and treatment group by timepoint interaction. The mean increase from baseline to each timepoint was higher in the IPN-20-SENSE LIDOCAINE arm than in the HA-RK-Lido arm (Table 4). However, this difference was statistically significant at 3 and 6 months only (9.3 [2.1; 16.5], *p*=0.012 and 10.5 [2.5; 18.6], *p*=0.011, respectively).

The blinded live evaluators’ GAIS assessments of participants’ improvement are presented in Tables 5 and S1. The improvement rate was higher in participants randomized to IPN-20-SENSE LIDOCAINE at all assessment timepoints and this was statistically significant at 6, 9 and 12 months.

**Table 5:** Global aesthetic improvement of the lips assessed by the blinded live evaluators at the different follow-up timepoints and in between groups comparative analysis—Secondary endpoint—Per-protocol population

Parameter	Month 1^a^		Month 3		Month 6		Month 9		Month 12	
	IPN-20-SENSE LIDOCAINE (*N*=44)	HA-RK-Lido (*N=*43)	IPN-20-SENSE LIDOCAINE (*N=*44)	HA-RK-Lido (*N=*43)	IPN-20-SENSE LIDOCAINE (*N=*44)	HA-RK-Lido (*N=*43)	IPN-20-SENSE LIDOCAINE (*N=*44)	HA-RK-Lido (*N=*43)	IPN-20-SENSE LIDOCAINE (*N=*44)	HA-RK-Lido (*N=*43)
n	44	43	42	41	42	39	39	36	43	40
*GAIS Improvemen*t										
Improved subjects^b^	41 (93.2%)	37 (86.0%)	42 (100.0%)	38 (92.7%)	42 (100.0%)	35 (89.7%)	39 (100.0%)	29 (80.6%)	39 (90.7%)	24 (60.0%)
[95% CI]^c^	[81.3; 98.6]	[72.1; 94.7]	[91.6; 100.0]	[80.1; 98.5]	[91.6; 100.0]	[75.8; 97.1]	[91.0; 100.0]	[64.0; 91.8]	[77.9; 97.4]	[43.3; 75.1]
Difference in improvement rate^d^	7.1		7.3		10.3		19.4		30.7	
[95% CI]^e^	[-8.1; 22.7]		[−4.4; 21.0]		[−2.3; 25.2]		[4.0; 36.6]		[10.6; 48.5]	
*p* value^f^	0.314		0.116		0.049		0.004		0.002	

*CI* Confidence interval; *GAIS* Global aesthetic improvement scale; *N* Number of subjects; *n* Number of subjects with available data

^a^Evaluation at Month 1 performed before the touch-up injection (if touch-up performed)

^b^Improvement on the 5-point GAIS corresponds to any of the three following categories “Very much improved”, “Much improved” and “Improved”

^c^Clopper–Pearson method

^d^Difference calculated as [change from baseline in the IPN-20-SENSE LIDOCAINE arm] minus [change from baseline in the HA-RK-Lido arm]

^e^Newcombe–Wilson method with continuity correction

^f^Fisher’s exact test

The mean (SD) pain scores reported by subjects at baseline and 1 month (if a touch-up was performed) were 4.2 (1.69) and 3.3 (1.68) for IPN-20-SENSE LIDOCAINE compared to 4.5 (1.91) and 4.2 (1.52) for HA-RK-Lido, respectively. These differences were not statistically significant (*p*=0.311 at baseline and *p*=0.103 at touch-up).

### Safety Analysis

ISRs reported by investigators immediately after the initial injection (mainly redness/erythema and swelling/edema) were comparable in both study arms and none of them were considered severe and resolved spontaneously by 3 months after treatment initiation. At 6, 9 and 12 months after treatment initiation, the only ongoing ISRs were of mild intensity and consisted of lumps/bumps (up-to Month 9) and discoloration (1 event ongoing up-to Month 12, which was also reported as a treatment-emergent adverse device effect).

Based on subject-reported ISRs, the ISR having the longest mean duration was related to lumps/bumps for both treatment arms (29.36 [63.874] days in the IPN-20-SENSE LIDOCAINE and 26.62 [44.883] days in the HA-RK-Lido arm). The mean duration of the other ISRs ranged between 0.29 days (for subject-reported itching and product migration in the IPN-20-SENSE LIDOCAINE arm) and 7.27 days (for subject-reported discoloration in the IPN-20-SENSE LIDOCAINE arm). The mean duration of most ISRs reported by subjects were also comparable between the 2 treatment arms.

An overview of AEs and treatment-emergent adverse events (TEAEs) reported in the SMILE study are presented in Tables S2 and S3.

## Discussion

The difference between the treatment arms in the proportion of improved subjects was +7.0 [−2.2; 17.7]. The lower limit of this 90% CI being above the non-inferiority margin of −15 and below zero demonstrated the non-inferiority, but not the superiority, of IPN-20-SENSE LIDOCAINE vs. HA-RK-Lido. At 6 months, the aesthetic improvement difference between both devices was 15.8 [−0.8; 32.6] favoring IPN-20-SENSE LIDOCAINE. By 9 and 12 months the proportion of improved subjects decreased in both arms but was still higher in IPN-20-SENSE LIDOCAINE (86.0% vs. 76.7%). The results of the blinded live evaluators’ GAIS assessments confirmed those obtained following the subjects’ self-evaluations.

The treatment effect of both devices decreased over time, which can be explained by the resorbable nature of HA gels. Nonetheless, a more sustained effect was observed following treatment with IPN-20-SENSE LIDOCAINE. A similar pattern was also identified on the FACE-Q™ “Satisfaction with lips” questionnaire. The decrease in subjects’ and independent assessors’ GAIS and FACE-Q™ “Satisfaction with Lips” scores was expected in view of the resorbable nature of HA fillers. Previous studies where GAIS was used for the evaluation of aesthetic improvement of the lips following HA fillers injection have also reported high proportions of improved subjects after treatment that decreased gradually over time [8, 9]. Similar trends in relation to FACE-Q™ “Satisfaction with lips” scores following treatment with HA-RK-Lido have been observed previously, with peak scores reported at 8 weeks after treatment initiation and lower scores reported at 48 weeks, but still higher than the baseline [9].

It is interesting to note that these results were obtained by comparing the clinical effects induced by two products that are relatively similar in terms of composition, as well as theoretical rheological behavior. Thus, the values of *G*’, *G*’’ and tan δ are, probably, not the only factors to consider when evaluating the performance of a product intended for lips treatment. Indeed, this indication is very specific as anatomical structures are highly mobile. Therefore, subjective evaluation of the product’s performance should not only be limited to the intensity of tissue projection. However, it should also consider the naturalness of the result obtained. It is plausible that the different cross-linking techniques used during the products’ manufacturing processes may have a significant impact on their ability to integrate within the tissues, especially during intense mobility sequences [11].

There were no overt differences that could be noted between the two arms with regard to the device safety and tolerability. The ISRs and adverse device effects observed were all expected events according to the devices’ instructions for use and most of which were mild and resolved spontaneously within 7 days. The proportions of some of the reported ISRs were slightly higher in the IPN-20-SENSE LIDOCAINE group, which could be related to the slightly higher mean volume and number of injection points in this cohort compared to the active control. The safety results obtained in the SMILE study are consistent with the results reported in a previous study in which HA fillers (including HA-RK-Lido) were used into and/or around the lips [8, 9, 12]. These results confirm that IPN-20-SENSE LIDOCAINE has a good safety profile comparable to that of HA-RK-Lido. The safety of the latter device had been well-established and demonstrated in several randomized controlled trials [8, 9].

The main strength of the SMILE study lies in its design. The risk of bias has been mitigated by ensuring that subjects and evaluators were blind to the injected device. Furthermore, involvement of several injectors from different countries increases the generalizability and reproducibility of these findings. We also used a comprehensive set of patient-reported and clinicians-reported core outcomes were assessed at different timepoints. However, there are some limitations to the study. These were mainly related to the lack of availability of quantitative scales or objective measures for the assessment of lips volume augmentation or redefinition, and hence, only subjective evaluations were used. Nevertheless, in the field of aesthetics, patient-reported results are as important as objective measurements in assessing the success of aesthetic treatment. Indeed, GAIS was used in several other comparable studies [8, 9, 13, 14]. Furthermore, the effectiveness of IPN-20-SENSE LIDOCAINE without lidocaine was previously demonstrated using objective measures [14].

## Conclusion

The SMILE study demonstrated the non-inferiority of IPN-20-SENSE LIDOCAINE to HA-RK-Lido in the aesthetic improvement of the lips based on subjects reported aesthetic improvement 3 months after the first injection. These findings were supported by the results of the blinded independent assessors. However, the effect of both treatments decreased over time, which was expected given the resorbable nature of HA fillers. Nonetheless, aesthetic improvement was sustained longer in the IPN-20-SENSE LIDOCAINE arm. Pain felt during the injection of both devices was tolerable for most subjects, and all injection site reactions and device-related adverse events reported in this study were expected and already described. Therefore, both IPN-20-SENSE LIDOCAINE and HA-RK-Lido offer a positive risk-benefit ratio up-to 12 months after treatment initiation and this is, broadly, equivalent between both products.

## Supplementary Information

Below is the link to the electronic supplementary material.

Supplementary file1 (DOCX 39 KB)
