# Supplementary material for: Effectiveness and Safety of IPN-20-SENSE LIDOCAINE for Lip Volume Augmentation and/or Redefinition (SMILE Study): A Non-inferiority Randomized Double-Blinded Controlled Study
Source: Aesthetic Plast Surg. 2024 Oct 14;49(4):1033–45. doi: 10.1007/s00266-024-04398-z (PMC11893706; doi:10.1007/s00266-024-04398-z)
Supplement: Supplementary file 1 — Supplementary file1 (DOCX 39 KB) [file 266_2024_4398_MOESM1_ESM.docx]

**List of Inclusion / Exclusion criteria**

Main inclusion criteria:

- Healthy subjects, male/female, between 18 and 65 years old.
- Subjects having given freely and expressly their informed consent and data privacy consent.
- Subject who expressed the wish for augmentation or restoration of the volume of the lips and/or lips’ outline redefinition with attainable expectations.
- Lips requiring volume and/or lips’ outline requiring redefinition according to the investigators’ judgement.
- Female of childbearing potential under a medically accepted contraceptive regimen since at least 12 weeks before being included in the study and during all the study follow-up period.

Main exclusion criteria:

- Pregnant or breastfeeding women or planning a pregnancy during the study.
- Subjects who could not provide full and free consent, enrolled in other clinical trials.
- Subjects with scars, moles or anything in the studied zones which might interfere with the evaluation.
- Subjects with severe perioral wrinkles.
- Subjects with contraindication to the use of products or medical conditions that the healthcare professional deemed inappropriate for the trial (e.g. allergy, autoimmune disease, hemostatic/bleeding disorders, infection, epilepsy, porphyria, severe impaired cardiac, hepatic and renal function).
- Subjects suffering from a severe or progressive disease or any other pathology that may interfere with the evaluation of the study results and/or subject safety.
- Subjects predisposed to keloidosis, hypertrophic scarring or healing disorders.
- Subjects with a known hypersensitivity or allergy to one of the ingredients of the tested products (e.g. hyaluronic acid, lidocaine or amide-type local anesthetic).
- Subjects suffering from inflammatory and/or infectious cutaneous disorders on or around the lips (herpes, acne…).
- Subjects with a recent history of facial injections, permanent or semi-permanent filling products, and cosmetic facial surgery or skin treatments (within the past 12 to 18 months prior to inclusion).
- Subjects who received oral surgery within 6 weeks prior to inclusion or who planned to undergo oral surgery during the study.

**Global Aesthetic Improvement Scale - rating system**

| Rating | | Description |
| --- | --- | --- |
| 1 | Very much improved | Optimal cosmetic result from injectable in this subject |
| 2 | Much improved | Marked improvement in appearance from the initial condition, but not completely optimal for this subject  Note: A touch-up would slightly improve the result |
| 3 | Improved | Obvious improvement in appearance from the initial condition  Note: A touch-up is indicated/advised |
| 4 | No change | The appearance is essentially the same as the original condition |
| 5 | Worse | The appearance is worse than the original condition |

The FACE-Q™  “Satisfaction with Lips” questionnaire is a validated questionnaire used in clinical practice, research, and quality improvement to incorporate cosmetic subjects’ perspective in outcome assessments (possible scores range between 0 and 100, with higher scores indicating greater satisfaction) The surface aspect of the lips questionnaire includes 5 items (“Volume repulped lips”, “Hydration Moisturized lips; lips’ softness/smoothness”, “Crumpled aspect of red lips”, “Contour/outline definition”, “Coloration/rosiness”), and 5 possible answers (“Much improved”, “Improved”, “No change”, “Worsened” and “Very worsened”) for each item.

**Injection site reaction scoring tool**

| **Parameter** | **None^a^** | **Mild^b^** | **Moderate^c^** | **Severe^d^** |
| --- | --- | --- | --- | --- |
| Redness/Erythema | _0_ | _1_ | _2_ | _3_ |
| Pain/Tenderness | _0_ | _1_ | _2_ | _3_ |
| Transient paresthesia^e^ | _0_ | _1_ | _2_ | _3_ |
| Induration | _0_ | _1_ | _2_ | _3_ |
| Swelling/Edema | _0_ | _1_ | _2_ | _3_ |
| Lumps/Bumps | _0_ | _1_ | _2_ | _3_ |
| Bruising | _0_ | _1_ | _2_ | _3_ |
| Itching | _0_ | _1_ | _2_ | _3_ |
| Coloration | _0_ | _1_ | _2_ | _3_ |
| Discoloration | _0_ | _1_ | _2_ | _3_ |
| Product migration | _0_ | _1_ | _2_ | _3_ |
| ^a^ None: the sign is not present.  ^b^ Mild: discomfort is present, but does not cause a disruption in the subject’s daily activities.  ^c^ Moderate: discomfort, leading to a reduction or affecting the subject’s daily activities.  ^d^ Severe: discomfort preventing the subject from working and/or pursuing the daily activities.  ^e^ In the subjects’ diaries, “transient paresthesia” was replaced by “transitory sensitivity trouble” for an easier comprehension.  Note: This scoring tool was completed by the subjects on a daily basis, during 1 month after each injection (initial injection, and touch-up [if performed]). | | | | |

The ISR scoring tool included potential events reported in the instructions for use of IPN-20-SENSE LIDOCAINE and HA-RK-Lido. ISRs were only reported as a device-related AEs (i.e., Adverse Device Effects) if a concomitant treatment was required, if the event needed follow-up by the evaluator or if the event was still present one month after the first injection regardless of the actual ISR’s start date.

**Injections procedure:**

Before the injection, the area to be treated was disinfected with an appropriate antiseptic solution and left to dry. After the injection, the injected area was well massaged to optimize the uniformity of the correction and subjects were asked to follow the recommendations in the instructions for use of the devices, which included not wearing make-up for the 12 hours after the injection, avoiding exposure of the treated areas to heat, sun or to extreme cold ( at least until any possible post‑injection swelling or redness or any other signs of local inflammation have disappeared), not applying a cold pack to the lips, using a high sun protection factor sunscreen on the lips if exposed to the sun for 2 weeks after the injection, and informing the investigational site of any “abnormal” developments in the treated areas. For each participant, the maximum authorized total volume of injected product for both lips combined was 2 mL. The exact volume to be injected to achieve optimal aesthetic result was determined by the injector. The techniques used for injection were determined by the injector based on their own experience/practice and on the subject’s expectations, and in line with the techniques recommended for both study devices (linear threading, multipoint/serial puncture or combination of techniques). Depending on the injector’s preference, the injection was performed either with the 30G½” needles provided with the devices or with 25G cannulas supplied for the purpose of this study. Participants were invited back for follow-up appointments at 1, 3, 6, 9 and 12 months post-injection when the primary and secondary outcomes were collected. An optional touch-up was possible at the 1-month follow-up appointment if deemed necessary, according to the subject’s and injector’s opinions (maximum authorized total volume for both lips was 1 mL). Overall, the maximum recommended total volume of product injected, in total for both lips, was 2.5 mL. All study injectors were qualified practitioners (3 aesthetic surgeons in France and 2 dermatologists in Poland) with previous significant experience in the use of both study devices. Both the subjects and the live evaluators were blind to the injected device throughout the study. The evaluators used photographs of the subjects taken before the first injection as a reference for comparison and, the same evaluator followed‑up the same subjects throughout the study period.

***Injection characteristics***

At baseline, all 92 subjects included in the SMILE study were injected in the upper lip. The total mean (SD) volume of product injected in the upper lip was 0.694 (0.2196) mL and 0.605 (0.2168) mL in the IPN-20-SENSE LIDOCAINE and HA-RK-Lido arms respectively. The lower lip was injected in 86 (93.5%) subjects (43 in each arm), and the mean volume of device injected was 0.334 (0.1584) mL and 0.276 (0.1334) mL in the intervention and active control arms respectively.

A total of 48 (53.3%) subjects had a touch-up for either the upper lip, lower lip or both lips at the 1-month follow-up visit. Of these, 21 (47.7%) subjects were in the IPN-20-SENSE LIDOCAINE arm and 27 (58.7%) subjects in the HA-RK-Lido arm. Considering both lips together for the touch-up injection, the mean (SD) injected volume of IPN-20-SENSE LIDOCAINE was 0.507 (0.2758) mL, and the mean (SD) injected volume of HA-RK-Lido was 0.554 (0.2568) mL.

Overall, the mean (SD) total volume of IPN-20-SENSE LIDOCAINE injected into both lips was comparable to the mean (SD) total volume of HA-RK-Lido injected into both lips when both initial and touch-up injections were considered together (1.250 (0.4878) mL and 1.176 (0.4312) mL, respectively).

The technique and materials used for injection were comparable in both study groups. In both treatment arms, the most frequently used injection techniques (in either lip) were the retrograde linear threading with the cannula and the multipoint injection/serial puncture with the needle.

**Table S1. Global Aesthetic Improvement Scale, according to the blinded independent evaluators and to the subjects themselves at all timepoints - Per‑protocol population**

| Parameter | Month 1 ^a^ | | Month 3 | | Month 6 | | Month 9 | | Month 12 | |
| --- | --- | --- | --- | --- | --- | --- | --- | --- | --- | --- |
|  | **STYLAGE^®^ M Lidocaïne (N=44)** | **Restylane^®^ Kysse^™^ (N=43)** | **STYLAGE^®^ M Lidocaïne (N=44)** | **Restylane^®^ Kysse^™^ (N=43)** | **STYLAGE^®^ M Lidocaïne (N=44)** | **Restylane^®^ Kysse^™^ (N=43)** | **STYLAGE^®^ M Lidocaïne (N=44)** | **Restylane^®^ Kysse^™^ (N=43)** | **STYLAGE^®^ M Lidocaïne (N=44)** | **Restylane^®^ Kysse^™^ (N=43)** |
| GAIS categories – Subjects | | | | | | | | | | |
| n | 44 | 43 | 44 | 43 | 42 | 39 | 43 | 41 | 43 | 40 |
| Very much improved | 15 (34.1%) | 8 (18.6%) | 14 (31.8%) | 8 (18.6%) | 15 (35.7%) | 4 (10.3%) | 9 (20.9%) | 7 (17.1%) | 6 (14.0%) | 5 (12.5%) |
| Much improved | 11 (25.0%) | 10 (23.3%) | 20 (45.5%) | 12 (27.9%) | 15 (35.7%) | 15 (38.5%) | 15 (34.9%) | 8 (19.5%) | 6 (14.0%) | 7 (17.5%) |
| Improved | 18 (40.9%) | 20 (46.5%) | 10 (22.7%) | 20 (46.5%) | 10 (23.8%) | 12 (30.8%) | 13 (30.2%) | 14 (34.1%) | 21 (48.8%) | 12 (30.0%) |
| No change | 0 (0.0%) | 5 (11.6%) | 0 (0.0%) | 3 (7.0%) | 2 (4.8%) | 8 (20.5%) | 6 (14.0%) | 12 (29.3%) | 10 (23.3%) | 16 (40.0%) |
| Worse | 0 (0.0%) | 0 (0.0%) | 0 (0.0%) | 0 (0.0%) | 0 (0.0%) | 0 (0.0%) | 0 (0.0%) | 0 (0.0%) | 0 (0.0%) | 0 (0.0%) |
| GAIS categories – Investigators (blinded independent evaluators, in live) | | | | | | | | | | |
| n | 44 | 43 | 42 | 41 | 42 | 39 | 39 | 36 | 43 | 40 |
| Very much improved | 1 (2.3%) | 2 (4.7%) | 5 (11.9%) | 1 (2.4%) | 1 (2.4%) | 0 (0.0%) | 0 (0.0%) | 0 (0.0%) | 0 (0.0%) | 0 (0.0%) |
| Much improved | 14 (31.8%) | 9 (20.9%) | 19 (45.2%) | 15 (36.6%) | 14 (33.3%) | 5 (12.8%) | 10 (25.6%) | 3 (8.3%) | 3 (7.0%) | 1 (2.5%) |
| Improved | 26 (59.1%) | 26 (60.5%) | 18 (42.9%) | 22 (53.7%) | 27 (64.3%) | 30 (76.9%) | 29 (74.4%) | 26 (72.2%) | 36 (83.7%) | 23 (57.5%) |
| No change | 3 (6.8%) | 6 (14.0%) | 0 (0.0%) | 3 (7.3%) | 0 (0.0%) | 4 (10.3%) | 0 (0.0%) | 7 (19.4%) | 4 (9.3%) | 15 (37.5%) |
| Worse | 0 (0.0%) | 0 (0.0%) | 0 (0.0%) | 0 (0.0%) | 0 (0.0%) | 0 (0.0%) | 0 (0.0%) | 0 (0.0%) | 0 (0.0%) | 1 (2.5%) |
| GAIS=Global Aesthetic Improvement Scale; N=Number of subjects; n=Number of subjects with available data.  *^a^ Evaluation at Month 1 performed before the touch‑up injection (if touch-up performed).*  *^b^ Improvement on the 5-point GAIS corresponds to any of the three following categories “Very much improved”, “Much improved” and “Improved”.* | | | | | | | | | | |

**Table S2. Overview of adverse events and treatment-emergent adverse events – Safety population**

| **Category** | **IPN-20-SENSE LIDOCAINE (N=45)** |  | | **HA-RK-Lido**  **(N=47)** |
| --- | --- | --- | --- | --- |
|  | n (%) m | | n (%) m | |
| **Any AE** | **28 (62.2%) 115** | | **31 (66.0%) 89** | |
| **TEAE** | **28 (62.2%) 112** | | **30 (63.8%) 86** | |
| Mild TEAEs | 28 (62.2%) 108 | | 29 (61.7%) 77 | |
| Moderate TEAEs | 4 (8.9%) 4 | | 6 (12.8%) 9 | |
| **Action taken following TEAEs** |  | |  | |
| TEAEs leading to corrective treatment | 24 (53.3%) 86 | | 20 (42.6%) 61 | |
| TEAEs preventing touch-up injection | 1 (2.2%) 1 | | 1 (2.1%) 1 | |
| **Outcome** |  | |  | |
| Ongoing | 3 (6.7%) 3 | | 0 (0.0%) 0 | |
| Resolved | 28 (62.2%) 109 | | 30 (63.8%) 86 | |
| **Relationship of TEAE to the device** |  | |  | |
| Not related to the device | 24 (53.3%) 91 | | 21 (44.7%) 64 | |
| Related to the device | 18 (40.0%) 21 | | 17 (36.2%) 22 | |
| Causal relationship | 14 (31.1%) 16 | | 13 (27.7%) 16 | |
| Possibly related | 4 (8.9%) 5 | | 5 (10.6%) 6 | |
| **TEAEs related to the device** |  | |  | |
| Mild TEAEs related to the device | 18 (40.0%) 21 | | 15 (31.9%) 18 | |
| Moderate TEAEs related to the device | 0 (0.0%) 0 | | 3 (6.4%) 4 | |
| **Related TEAEs following an ISR ^a^** |  | |  | |
| Yes | 16 (35.6%) 18 | | 16 (34.0%) 20 | |
| No | 3 (6.7%) 3 | | 1 (2.1%) 2 | |
| **Action taken following TEAEs related to the device** |  | |  | |
| Related TEAE leading to corrective treatment | 1 (2.2%) 1 | | 1 (2.1%) 2 | |
| Related TEAE preventing touch-up injection | 1 (2.2%) 1 | | 1 (2.1%) 1 | |
| None | 11 (24.4%) 12 | | 12 (25.5%) 14 | |
| Other | 6 (13.3%) 8 | | 4 (8.5%) 6 | |
| **Outcome** |  | |  | |
| Ongoing | 1 (2.2%) 1 | | 0 (0.0%) 0 | |
| Resolved | 17 (37.8%) 20 | | 17 (36.2%) 22 | |
| * **Safety (SAF) population**: comprised all the subjects who were included and who received at least one treatment with any of the study devices. Subjects in the SAF population were analyzed according to the received treatment.  AE=Adverse event; ISR=Injection site reaction; m=Number of events; N=Number of subjects; n=Number of subjects experiencing at least one event; TEAE=Treatment-emergent adverse event.  *^a^An ISR was considered as an AE if a concomitant treatment was taken for its treatment, if it was followed‑up by an Investigator, or if it was still present 1 month after the injection.* | | | | |

**Table S3. Treatment-Emergent Adverse Events Related to the Devices by Primary System Organ Class and Preferred Term - Safety Population**

|  | **IPN-20-SENSE LIDOCAINE (N=45)** | | **HA-RK-Lido (N=47)** | |
| --- | --- | --- | --- | --- |
|  | **n (%)** | **m** | **n (%)** | **m** |
| At least one treatment-emergent adverse event related to the devices | 18 (40.0%) | 21 | 17 (36.2%) | 22 |
| General disorders and administration site conditions | 15 (33.3%) | 17 | 16 (34.0%) | 20 |
| Injection site mass | 10 (22.2%) | 12 | 13 (27.7%) | 15 |
| Injection site dryness | 0 (0.0%) | 0 | 2 (4.3%) | 2 |
| Injection site discoloration | 1 (2.2%) | 1 | 0 (0.0%) | 0 |
| Injection site hematoma | 1 (2.2%) | 1 | 0 (0.0%) | 0 |
| Injection site hemorrhage | 1 (2.2%) | 1 | 0 (0.0%) | 0 |
| Injection site oedema | 0 (0.0%) | 0 | 1 (2.1%) | 1 |
| Injection site pain | 1 (2.2%) | 1 | 0 (0.0%) | 0 |
| Injection site papule | 1 (2.2%) | 1 | 0 (0.0%) | 0 |
| Injection site paresthesia | 0 (0.0%) | 0 | 1 (2.1%) | 1 |
| Sensation of blood flow | 0 (0.0%) | 0 | 1 (2.1%) | 1 |
| Infections and infestations | 2 (4.4%) | 3 | 1 (2.1%) | 1 |
| Oral herpes | 2 (4.4%) | 3 | 1 (2.1%) | 1 |
| Gastrointestinal disorders | 1 (2.2%) | 1 | 0 (0.0%) | 0 |
| Lip dry | 1 (2.2%) | 1 | 0 (0.0%) | 0 |
| Skin and subcutaneous tissue disorders | 0 (0.0%) | 0 | 1 (2.1%) | 1 |
| Skin tightness | 0 (0.0%) | 0 | 1 (2.1%) | 1 |

n: number of subjects with at least one TEAE related to the device, %: (n row / N column) x 100; m: number of TEAEs related to the device.

TEAEs related to the devices are those with at least one episode categorized by the investigator as 'probably related', 'possibly related' or 'causal relationship' to the device or with relationship to the device missing.

MedDRA dictionary version 24.0.

TEAEs considered related to the investigational device or to the active control device were reported with similar frequencies in each treatment arm. Most of these events were either associated with ISRs or were reported following an ISR, indicating that these events were expected with this kind of procedure and are already described in the “instructions for use” of the devices (**Tables S2 and S3**). The mean (SD) duration of device‑related TEAEs was comparable between the intervention and active control arms (69.3 [91.07] days and 65.3 [73.23] days, respectively). However, device‑related TEAEs tended to appear later following IPN-20-SENSE LIDOCAINE (mean [SD] time to onset since the first injection of 10.8 [32.84] days, and since the last injection of 9.4 [26.95] days) than following HA-RK-Lido (mean [SD] time to onset since the first injection of 3.9 [10.04] days, and since the last injection of 2.6 [8.49] days). No serious AEs and no device deficiencies were reported in either treatment arm throughout the study.
